# Salience network connectivity is altered in 6-week-old infants at heightened likelihood for developing autism

**DOI:** 10.1038/s42003-024-06016-9

**Published:** 2024-04-22

**Authors:** Tawny Tsang, Shulamite A. Green, Janelle Liu, Katherine Lawrence, Shafali Jeste, Susan Y. Bookheimer, Mirella Dapretto

**Affiliations:** 1 Meta, Menlo Park, CA USA; 2 Ahmanson-Lovelace Brain Mapping Center, University of California, Los Angeles, Los Angeles, CA USA; 3 Department of Psychiatry and Biobehavioral Sciences, University of California, Los Angeles, Los Angeles, CA USA; 4 Semel Institute of Neuroscience and Human Behavior, David Geffen School of Medicine, University of California, Los Angeles, Los Angeles, CA USA; 5 Center for Cognitive Neuroscience, University of California, Los Angeles, Los Angeles, CA USA; 6 Cedars Sinai Hospital, Los Angeles, CA USA; 7 Keck School of Medicine, University of Southern California, Los Angeles, CA USA; 8 Children’s Hospital Los Angeles, USC Keck School of Medicine, Los Angeles, CA USA

**Keywords:** Autism spectrum disorders, Sensory processing

## Abstract

Converging evidence implicates disrupted brain connectivity in autism spectrum disorder (ASD); however, the mechanisms linking altered connectivity early in development to the emergence of ASD symptomatology remain poorly understood. Here we examined whether atypicalities in the Salience Network – an early-emerging neural network involved in orienting attention to the most salient aspects of one’s internal and external environment – may predict the development of ASD symptoms such as reduced social attention and atypical sensory processing. Six-week-old infants at high likelihood of developing ASD based on family history exhibited stronger Salience Network connectivity with sensorimotor regions; infants at typical likelihood of developing ASD demonstrated stronger Salience Network connectivity with prefrontal regions involved in social attention. Infants with higher connectivity with sensorimotor regions had lower connectivity with prefrontal regions, suggesting a direct tradeoff between attention to basic sensory versus socially-relevant information. Early alterations in Salience Network connectivity predicted subsequent ASD symptomatology, providing a plausible mechanistic account for the unfolding of atypical developmental trajectories associated with vulnerability to ASD.

## Abstract (web summary)

Infants with heightened vs. lower likelihood of developing autism spectrum disorder exhibit different functional connectivity patterns in the salience network. These patterns predict sensory and social behaviors at 1 year of age.

## Introduction

Shortly after birth, newborns display systematic preferences for faces^1^, voices^2^, and biological motion^3^. These early social-orienting mechanisms are foundational for normative social development^4^. However, the salience of socially-relevant information appears disrupted in autism spectrum disorders (ASD). Infants who develop ASD show altered developmental trajectories^5^ characterized by reduced attention to social information^6,7^ and heightened awareness of non-social sensory input^8^. Atypicalities in social versus nonsocial attention are broadly recognized as a marker of increased likelihood for ASD^9^ and likely contribute to the emergence of social impairments and the restrictive and repetitive behaviors characteristic of ASD^7,10^. The neurobiological mechanisms underlying these early attentional abnormalities that give rise to autism-related symptoms remains largely unknown.

Examining early brain connectivity offers a promising lens for investigation^11,12^. Indeed, most genes associated with increased likelihood for ASD impact synapse formation and function, presenting a biological pathway for ASD that converges on brain connectivity^13,14^. Neuroimaging studies have consistently implicated atypical brain network dynamics in ASD^15,16^, with recent evidence demonstrating that 6-month-olds who later develop ASD already exhibit systematic differences in whole-brain functional connectivity^17^. Thus, early deviations in brain network connectivity may provide a biomarker of ASD likelihood prior to the emergence of behavioral symptoms^18^.

One early emerging functional brain network^19^ of particular interest in understanding ASD symptomatology is the Salience Network (SN)^20^, which is believed to be integral in guiding attention to the most salient interoceptive and exteroceptive stimuli^20,21^. Altered Salience Network connectivity can discriminate children with ASD from neurotypical controls with high classification accuracy^22^, and is associated with symptoms of restrictive/repetitive behaviors^22^, including atypical sensory processing^23^, which may highlight a potential neural mechanism underlying increased perceived salience of low-level, perceptual contingencies in the environment at the expense of higher-level social information^24^. The Salience Network has been identified in neonates^19,25,26^, and while not yet fully mature^19^, even in its early forms, it is thought to play a key role in influencing early brain development^27^. Furthermore, hypoconnectivity within Salience Network hubs has recently been reported in a small sample of neonates at increased likelihood for developing ASD based on family history^28^.

In addition to these consistent findings of atypical Salience Network connectivity in ASD, behavioral evidence also implicates this network in the emergence of ASD symptomatology in early infancy. Faces represent a highly salient class of stimuli for typically-developing infants^29^ and normative patterns of early Salience Network connectivity may support the initial attentional bias toward faces as well as subsequent developmental increases in visual attention to faces in the first year^30^. Conversely, early disruptions in Salience Network connectivity may iteratively derail processes that typically reinforce the perceived salience of faces^31^ by conferring heightened salience to lower-level non-social stimuli^24^. Indeed, initial overt symptom-based markers of ASD suggest deviations in processes that typically guide social communicative development, including attention to faces^5^ and speech. Potential atypicalities in Salience Network connectivity may thus contribute to the emergence of characteristic features of ASD (i.e., social communicative impairments and altered sensitivity to sensory stimuli).

To test this model, we evaluated Salience Network connectivity in 6-week-old infants, at high (HL) and typical (TL) likelihood of developing ASD, based on family history, and its association with subsequent behavioral ASD symptom-based markers, including atypicalities in visual social attention to faces, communicative development, and sensory processing. Between 4 and 8 weeks, early social-orienting behaviors transition from being under reflexive subcortical control to experience-dependent cortical control^32,33^. Thus, examining Salience Network connectivity during this period may reveal altered development of social attention and provide a mechanistic account for the ontogeny of ASD symptomatology. We hypothesized that HL infants would show Salience Network hyperconnectivity with sensorimotor regions^22,23^ and that patterns of Salience Network connectivity would predict individual trajectories of social attention, communicative development, and sensory sensitivities.

This study prospectively evaluated 53 HL and TL infants. HL infants had at least one older sibling with an ASD diagnosis (*N* = 24) whereas TL infants (*N* = 29) had no family history of ASD or any other developmental disorders. Infants underwent resting-state functional magnetic resonance imaging (rs-fMRI) at 6 weeks of age during natural sleep to examine Salience Network connectivity. Infants’ eye movements were tracked at 3-, 6-, 9-, and 12-months of age while they viewed video stimuli of naturalistic social interactions (i.e., excerpts from *Charlie Brown* and *Sesame Street*^34^) to capture developmental trajectories in visual social attention to faces. We examined core autism symptomatology with the Autism Observation Scale for Infants (AOSI) and nonverbal social-communication with the Early Social Communication Scale (ESCS) at 12 months (see Table 1), as well as sensory sensitivity with the Infant/Toddler Sensory Profile (ITSP) from 6- to 12-months.

**Table 1:** Participant demographics

		TL *N* = 29		HL *N* = 24		
		*N*	%	*N*	%	*P*
Sex						0.38
Female		11	37.93	17	58.62	
Male		18	62.07	12	41.38	
Race						0.88
White		20	68.97	17	58.62	
Non-white		9	31.03	12	41.38	
Family Income						0.23
Not Answered		1	3.45	0	0.00	
<50 K		3	10.34	6	25.00	
50–75 K		4	13.79	4	16.67	
75–100 K		5	17.24	5	20.83	
100–125 K		4	13.79	2	8.33	
>125 K		12	41.38	7	29.17	
		Mean	(SD)	Mean	(SD)	*P*
Birth Weight						
Pounds		7.65	1.67	7.41	2.43	0.67
Age at Scan						
Weeks		6.63	1.37	6.65	1.09	0.95
Relative Motion						
mm		0.23	0.82	0.10	0.07	0.44
Behavioral Scores at 12 months						
Mullen ELC		110.22	11.83	106.67	17.24	0.39
ESCS IJA		0.71	0.45	0.96	0.48	0.06
ESCS RJA		0.31	0.23	0.28	0.24	0.56
AOSI Total Score		4.12	1.83	4.71	3.07	0.97
Sensory Sensitivity		19.52	4.39	20.7	7.55	0.54

## Results and discussion

### Within- and between-group salience network connectivity

**Fig. 1:**
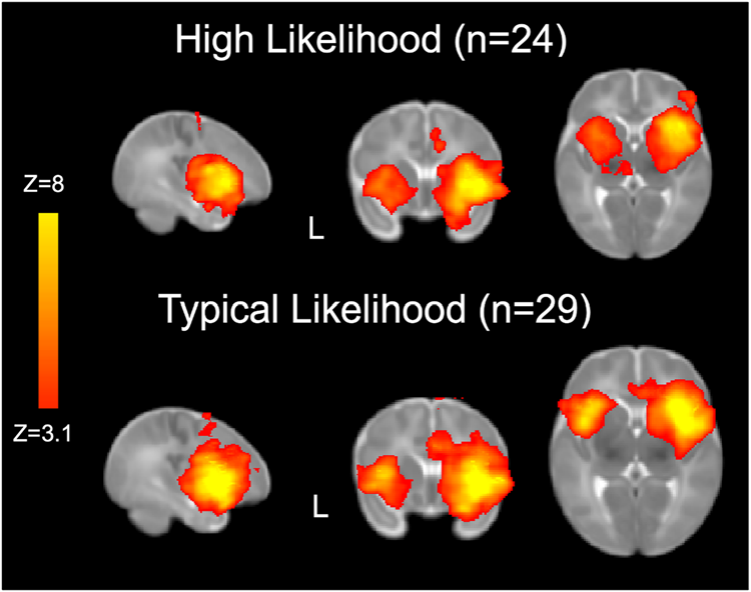
Salience Network connectivity. Robust Salience Network connectivity was detected in both 24 High Likelihood and 29 Typical Likelihood infants using the right anterior insula as the seed.

**Fig. 2:**
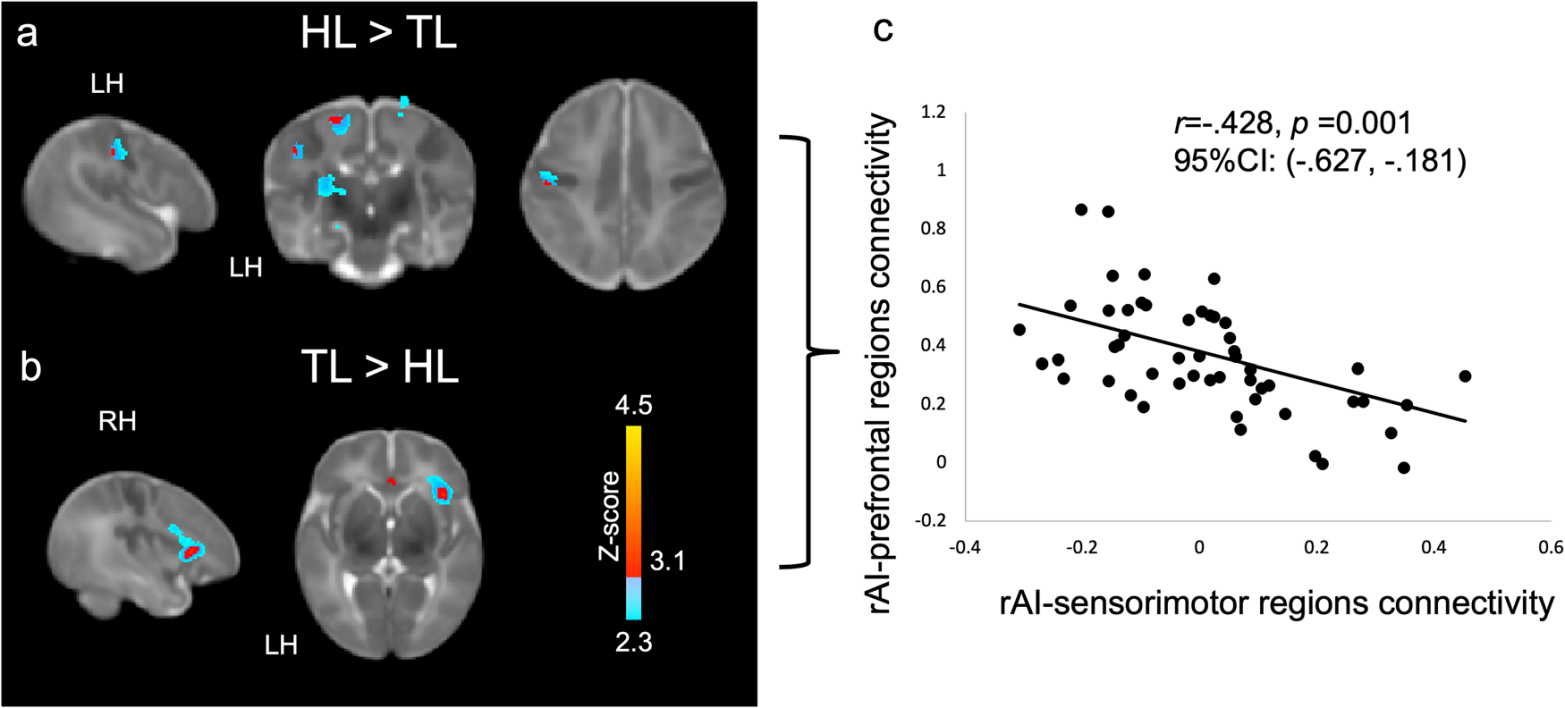
Between-group differences in Salience Network connectivity. **a** Relative to 29 Typical Likelihood (TL) infants, 24 High Likelihood (HL) infants showed greater right anterior insula (rAI) connectivity with left pre- and post-central gyri, thalamus, and caudate—regions associated with sensory and motor processing. **b** In contrast, TL infants show greater right anterior insula connectivity with right orbitalfrontal cortex and inferior frontal gyrus—frontal regions associated with social processing. **c** Parameter estimates of connectivity extracted from clusters showing significant between-group differences in Salience Network connectivity (HL > Tl and TL > HL) revealed that Salience Network connectivity patterns in these regions were inversely related to one another such that, across all 53 infants, greater connectivity between the right anterior insula and sensory regions was associated with weaker connectivity between right anterior insula and frontal, higher-order cognitive processing regions.

Consistent with prior work, we used a right anterior insula (rAI) seed to characterize Salience Network connectivity across the whole brain^23,35^ (Fig. 1). We first examined between-group differences in Salience Network connectivity based on the likelihood of developing ASD (Fig. 2). Compared to TL infants, HL infants showed stronger connectivity between the hub of the Salience Network (i.e., the rAI) and sensorimotor regions, including bilateral precentral gyrus and left postcentral gyrus, thalamus, hippocampus, caudate and putamen (Fig. 2a). In contrast, compared to TL infants, HL infants showed weaker connectivity between the rAI and prefrontal regions associated with higher-order processing and attentional control, including right inferior and middle frontal gyri, and anterior cingulate (Fig. 2b). Importantly, we found an inverse relationship in Salience Network connectivity with sensorimotor and prefrontal regions across all participants (*r* = *−0.43, p* = 0.001, 95% CI: 0.18–0.63) such that infants exhibiting the strongest Salience Network connectivity with sensorimotor regions also showed the weakest connectivity with higher-order prefrontal regions (Fig. 2c). This direct tradeoff between neural resources allocated towards sensorimotor processing versus social attention could thus explain the co-emergence of both core sensory and social ASD symptoms.

### Salience Network connectivity and developmental trajectories in attention to faces

**Fig. 3:**
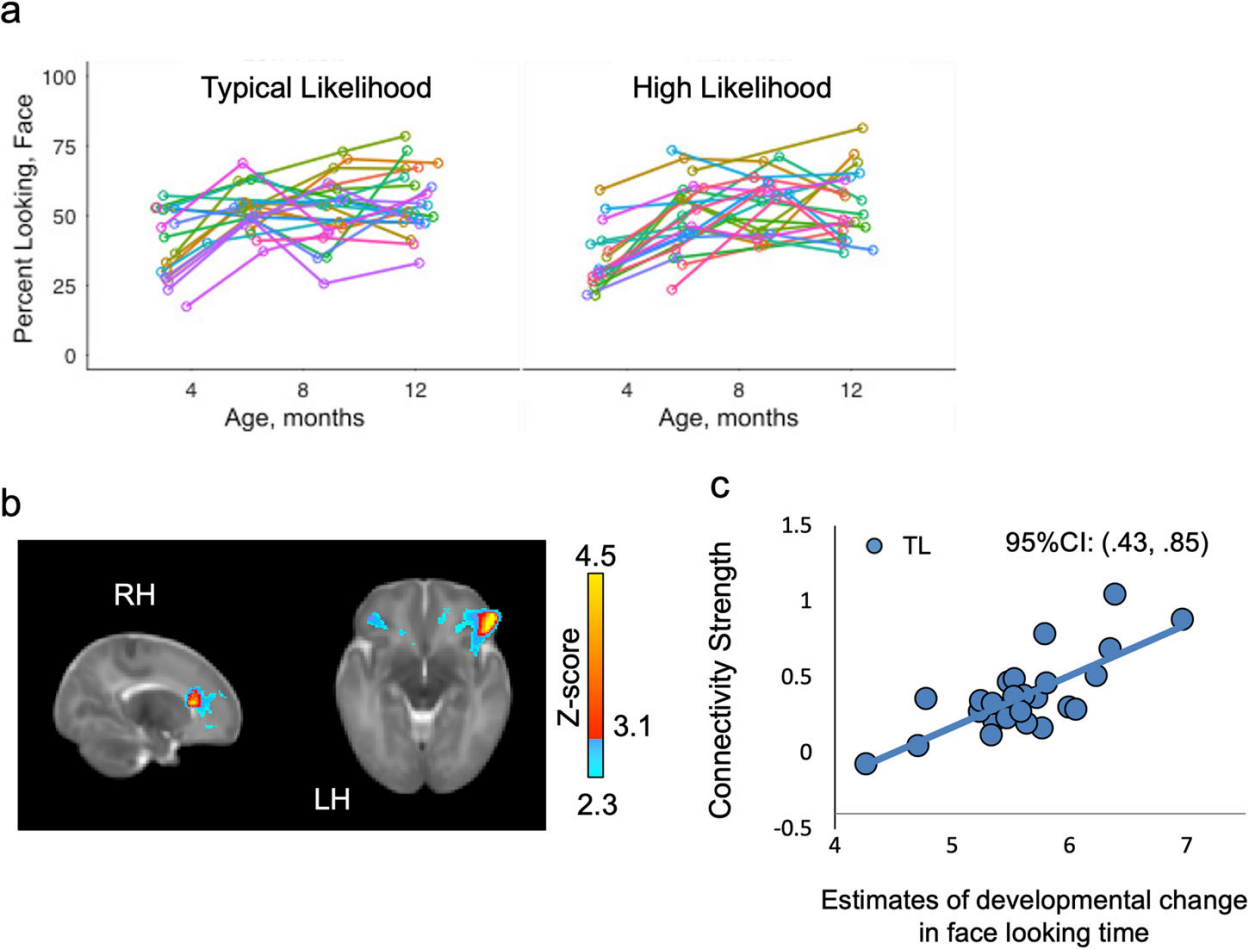
Salience Network connectivity and developmental trajectories in attention to faces. **a** Each infant’s raw percent looking at faces across time points are plotted in **a**, with different colors depicting different individual participants. Individual data points were analyzed in a Bayesian hierarchical linear model. Estimates of each infant’s change in looking time to faces across age was derived from the best-fit model and included as a regressor of interest in a linear model of Salience Network connectivity. **b** In 26 Typical Likelihood infants, greater right anterior insula connectivity with anterior cingulate cortex and right lateral orbitofrontal cortex was associated with greater increases in attention to faces from 3-to12-months of age. **c** Parameter estimates of connectivity from clusters shown in **b** are plotted against estimates of change in face-looking time across age.

This inverse pattern of connectivity between the Salience Network and social vs. sensorimotor regions indicates an early neural mechanism that may lead to divergent developmental outcomes for HL infants. To then directly examine the downstream, developmental effects of Salience Network connectivity on visual social attention to faces, which has been shown to be attenuated in HL infants^6^, we evaluated whether early Salience Network connectivity predicts individual trajectories in attention to faces from 3- to 12-months. We measured percent looking time to faces from the eye-tracking data (Fig. 3a) and used a Bayesian hierarchical linear model to estimate each infant’s rate of increased attention to faces from 3- to 12-months. This estimate was then used as a covariate of interest in our analysis of Salience Network connectivity.

HL infants did not show any significant associations between 6-week Salience Network connectivity and subsequent trajectories in attention to faces. In TL infants, however, greater connectivity between the two major hubs of the SN – rAI and the anterior cingulate cortex^20^ – and right lateral orbitofrontal cortex at 6 weeks predicted greater increases in attention to faces across the first postnatal year (Fig. 3b, c). Importantly, these frontal regions predicting increased social attention overlapped with those exhibiting greater Salience Network connectivity in TL infants relative to HL infants. Early social behavior, including orienting to faces and preferential attention to biological motion, is highly phylogenetically-conserved^3^ and genetically constrained^36^. Similarly, developmental changes in Salience Network connectivity during the first postnatal year appears to be strongly influenced by genetic effects, more so than environmental factors^37^. In light of our findings, strong Salience Network connectivity with frontal regions at 6 weeks may thus contribute to the early perceived salience of faces in typical development and thereafter support normative gains in social attention through iterative processes that further consolidate brain-behavior connections.

### Salience Network connectivity and later social- and sensory-processing development

**Fig. 4:**
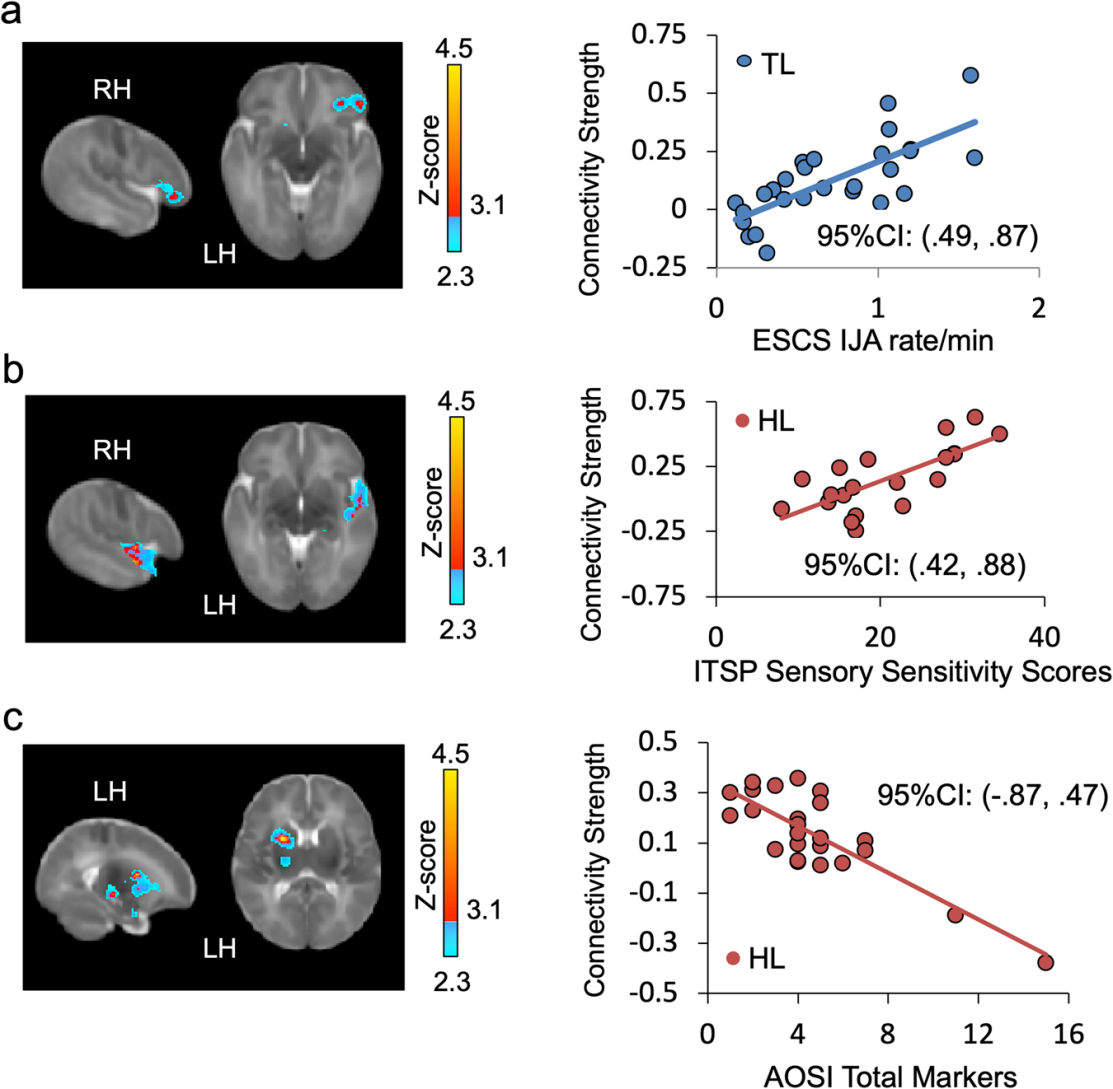
Associations between early Salience Network connectivity and later social and sensory-processing development. **a** Greater right anterior insula connectivity with right orbitofrontal cortex, reward-processing regions, and prefrontal regions predicted greater rates of initiating joint attention in 27 Typical Likelihood (TL) infants at 12months (ESCS IJA = Early Social Communication Skills, initiating joint attention). **b** Greater right anterior insula connectivity right superior temporal gyrus, amygdala and thalamus at 1.5 months predicted greater level of parent-reported sensory processing atypicalities in 24 High Likelihood (HL) infants (ITSP = Infant Toddler Sensory Checklist). **c** Greater right anterior insula connectivity with left basal ganglia, thalamus, and amygdala at 1.5 months predicted lower level of social impairment on the AOSI at 12 months in 24 HL infants (AOSI Autism Observation Scale for Infants).

We next examined the relation between Salience Network connectivity and standardized measures of communicative development (ESCS), ASD-related symptoms (AOSI), and sensory processing atypicalities (ITSP). In TL infants, greater connectivity between the Salience Network hub (i.e., rAI) and both prefrontal (i.e., right inferior frontal gyrus, middle frontal gyrus, and orbital frontal cortex) and subcortical regions associated with reward and learning (i.e., bilateral caudate, pallidum, and nucleus accumbens) predicted higher rates of initiating joint attention – nonverbal communicative behaviors associated with better language functioning and social competence^38^– at 12 months (Fig. 4a). Salience Network connectivity at 6 weeks was not related to response to joint attention in either group. This finding provides further evidence that normative patterns of Salience Network connectivity in early infancy support attention to socially relevant stimuli, thereby scaffolding the development of social communication skills. The lack of similar relationships between Salience Network connectivity and later social communication skills in the HL group could reflect the overall reduced Salience Network connectivity with frontal cortex at 6 weeks in this group. In other words, it is possible that this group shows delayed development of the early neural precursors of social communication, but further longitudinal investigations of connectivity across the first year of life are necessary to determine whether this is a delayed or atypical trajectory.

Distinct patterns of 6-week Salience Network connectivity also predicted ASD-associated symptoms in HL infants at 6–12 months (Fig. 4b, c). Greater Salience Network connectivity with regions associated with primary auditory and sensory processing (right superior temporal gyrus and thalamus) predicted higher parental ratings of sensory hypersensitivity at 6–12 months. This finding is consistent with observed associations between Salience Network hyperconnectivity and sensory over-responsivity in older children with ASD^23^. HL infants may be predisposed to attend to extraneous sensory inputs quite early in development at the expense of socially relevant information. Furthermore, stronger connectivity between the Salience Network hub and regions involved in implicit learning and reward processing (left putamen, inferior frontal gyrus, caudate, amygdala), and memory (left hippocampus) predicted fewer ASD symptoms on the AOSI at 12 months. Better integration between the Salience Network and both reward and emotional systems (e.g., regions associated with the social brain^39^) may be protective for social cognitive development in HL infants; conversely, reduced Salience Network connectivity with these social brain regions in early infancy may negatively impact social learning.

Our data reveal dissociable patterns of Salience Network connectivity between 6-week-old TL and HL infants with cascading effects on brain-behavioral processes that may underlie both normative and atypical social communicative development. Detectable differences in functional connectivity in early infancy corroborate cytoarchitectural and genetic data implicating prenatal neural development as a neurobiological factor that increases the likelihood for ASD^40^. The observed hyperconnectivity between the hub of the Salience Network and sensorimotor regions in HL infants likely represents a developmental vulnerability that, given the heterogeneity of developmental trajectories associated with a family history of ASD, may be broadly associated with suboptimal outcomes. The observed tradeoff in functional connectivity with these areas versus higher-order prefrontal regions highlight that ASD-associated brain development manifests as differences of degree, not kind.

Taken together, these results provide clear empirical support for recent theoretical frameworks positing that initial deviations in attentional biases and/or sensorimotor processing may lead to the emergence of ASD-related behaviors by altering the experience-dependent brain changes that typically guide social development^41^. Furthermore, our findings demonstrate that atypical functional brain connectivity is present as early as 6 weeks of age, long before detectable differences in behavior are observed in infants and toddlers at high likelihood for developing ASD. This difference is notable because it suggests a protracted prodromal period for known ASD-associated symptoms to emerge, including early social difficulties, attentional difficulties, language delays, and socioemotional problems. While specific patterns of Salience Network connectivity within the HL and TL infants predicted distinct levels of social functioning and sensory processing, HL and TL infants exhibited comparable behavioral profiles at 12 months, consistent with prior behavioral studies^10^. By affecting attentional biases, initial differences in brain connectivity may become compounded over development through individual experiences, highlighting the role of experience in shaping brain-behavior relationships and the opportunity to intervene to prevent the crystallization of atypical social communicative development.

Examining early functional brain connectivity can inform the impact of genetic and environmental factors on subsequent development by highlighting the intermediary neural networks that ultimately underlie behavior. For instance, recent work has suggested that prenatal exposure to maternal inflammatory markers negatively influence early Salience Network connectivity in infants without a family history of ASD^25^, which suggests an intricate interplay between genetic and environmental factors in fine-tuning brain-behavior connections and contributing to the individual variability in early social orienting behaviors. While a primary aim of the current study was to elucidate the role of Salience Network connectivity in conferring vulnerability to ASD, infants with a family history of ASD are also more likely to exhibit atypical development, including subclinical ASD symptomatology (i.e., broader autism phenotype), speech/language delays, global developmental delay, and Attention Deficit Hyperactivity Disorder (ADHD) during school-age years^42^. Thus, the clinical implications of our findings extend beyond the likelihood of an ASD diagnosis, providing a testable model for examining how early Salience Network connectivity may be related to other suboptimal developmental outcomes that are associated with a family history of ASD. Although our modest sample size and the single timepoint for evaluating Salience Network connectivity are limitations in the current study, the overall results strongly suggest that atypical patterns of Salience Network connectivity may reflect a developmental vulnerability. This is a possibility that should be examined in large-scale longitudinal studies that heavily sample brain and behavioral measures during the first postnatal years.

In sum, our findings demonstrate that aberrant patterns of functional brain connectivity can be detected in infants at high likelihood for developing ASD shortly after birth, and that these alterations predict ASD-related symptomatology a year later. Identifying atypical brain connectivity in infancy may ultimately pave the way for early interventions that can effectively redirect attention to socially relevant inputs and/or reduce distress caused by aversive sensory inputs and thus promote optimal developmental outcomes.

## Methods

### Participants

Participants in this study were enrolled as part of a longitudinal project examining early brain-based markers of ASD during the first year, with data collected between December 2012 through November 2017. The Institutional Review Board (IRB) at the University of California, Los Angeles, approved all protocols associated with the project, and all enrolled participants had informed consent provided by their parent/legal guardian. All procedures complied with ethical regulations for vulnerable populations. Infants were assigned to ASD-likelihood cohorts based on family history: high-likelihood infants (HL) had at least one older sibling with a clinical ASD diagnosis whereas typical-likelihood infants (TL) had no family history of ASD or any other developmental disorder. Prior research showed that the recurrence likelihood for developing ASD is approximately 20% in HL infants^43^. High and typical likelihood families were broadly recruited in Los Angeles County through IRB-approved fliers posted in pediatrician offices, newspapers, magazines, community-based events, and the Center for Autism Research and Treatment at the University of California, Los Angeles. Additional recruitment strategies included IRB-approved messages aired on Pandora and local radio stations, and word-of-mouth. This ensured a broad sampling. The sample size was based on prior resting-state fMRI studies in infants. Based on effect sizes derived from these prior studies (e.g., Liu et al. and Damaraju et al.), a minimum sample size of 23 per group was sufficient to detect significant effects. Exclusionary criteria for both groups included: 1) indication of genetic or neurological conditions associated with ASD likelihood (e.g., fragile X syndrome, epilepsy, tuberous sclerosis), 2) significant perinatal insult or chronic medical conditions impacting development, 3) severe visual, hearing, or motor impairment, 4) non-English speaking parents, and 5) contraindication for MRI (e.g., metal implants). All participants were enrolled in the study prior to 6 weeks of age. HL and TL infants were matched by gender (Mann–Whitney *U* = 306, Z = 0.87 *p* = 0.38, *r* = 0.12), and birth weight (*t*(51) = 0.43, *p* = 0.67, Cohen’s *d* = 0.12), as well as ethnicity and family socio-economic status (race: Mann–Whitney *U* = 341.5*, p* = 0.88; household income: Mann–Whitney *U* = 282.5, *Z* = 1.21 *p* = 0.23, *r* = 0.166). Given the diverse demographic of Los Angeles County, the research sample was representative of the broader population of infants with and without familial history of ASD. HL and TL infants did not differ on cognitive development at 12 months according to the Mullen Scales of Early Learning—Early Learning Composite (HL_ELC_ Mean=106.67, SD = 17.24; TL_ELC_ Mean = 110.22, SD = 11.83; *t*(49) = 0.87, *p* = 0.39, Cohen’s *d* = 0.24; see Table 1).

A total of 53 infants (*N* = 24 HL, *N* = 29 TL) underwent functional magnetic resonance imaging (fMRI) during natural sleep at approximately 6 weeks of age (HL_age_ Mean = 6.63 weeks, SD = 1.37 weeks; TL_age_ Mean = 6.65 weeks, SD = 1.09 weeks, *t*(51) = 0.062, *p* = 0.95, Cohen’s *d* = 0.02). A subset of 51 infants (*N* = 24 HL, *N* = 27 TL) provided data from behavioral measures of social and cognitive development at 12 months (2 TL infants dropped out of the study). Of the 53 infants, 50 infants (*N* = 24 HL; 26 TL) provided longitudinal eye-tracking data at 3-, 6-, 9-, and 12-months of age. An additional 3 infants participated in the study but were not included in the analyses due to excessive head motion during scanning and/or scanner artifacts. No statistical methods were used to pre-determine sample size, but our sample size is larger than what has been reported in previous publications evaluating resting-state connectivity in early infancy^44–46^.

HL infants were all later-born children; the TL infants in our sample included both first-born (*N* = 16) and later-born children (*N* = 13). We did not observe differences in overall connectivity strength within the SN between first- and later-born TL infants (t(27) = 1.07, *p* = 0.318, Cohen’s d = 0.379), nor did we observe differences in behavioral measures of cognitive and social development at 12 months (*t’*s < 1.90, *p*’s > 0.06, Cohen’s *d* < 0.53). Therefore, we do not believe birth order would have had an effect on the findings presented here.

### Behavioral measures

Infants were administered a battery of behavioral assessments during the first postnatal year to measure socio-communicative, cognitive, and sensory development (see Table 1). At 12 months, infants’ developmental level was assessed with the Mullen Scales of Early Learning^47^, nonverbal social communicative behaviors (i.e., rates of initiating of and responding to joint attention cues—IJA and RJA respectively) with the Early Social Communication Scales (ESCS^48^), and early signs of ASD symptomatology with the Autism Observation Scale for Infants (AOSI^49^). Approximately 20% of the total ESCS sample was double coded for reliability. Coders were trained undergraduate research assistants who were blind to ASD family status and other study variables. Intraclass correlations (ICC; absolute agreement, single measures) indicated good reliability for IJA (ICC = 0.96) and RJA (ICC = 0.89). Behavioral measures were administered blind to the infant’s ASD likelihood status. Parents also completed the Infant/Toddler Sensory Profile^50^, a standardized questionnaire tracking their child’s sensitivity to sensory inputs and sensory-related difficulties, at 6, 9, and 12 months; the average raw score on the Sensory Sensitivity quadrant was calculated and used as a general metric of sensitivity to visual, auditory, and tactile stimuli.

### Eye-tracking protocol

Infants were eye-tracked at the 3-, 6-, 9- and 12-month visits while they were presented with two, 2-mintue full audiovisual video segments taken from a cartoon and live-action video; these video stimuli have been previously used in studies on visual social attention to faces in typically-developing infants^34,51^. Infants sat on their caregiver’s lap during the eye-tracking procedure at approximately 60 cm from the 65-cm video display monitor. Caregivers were explicitly instructed not to distract their infant’s attention from the screen during stimuli presentation.

Point-of-gaze data were collected using a Tobii T60XL eye-tracker at 60 Hz with a spatial accuracy of approximately 0.5° accuracy. Eye-movements (e.g., fixations, blinks, and saccades) were detected using the accompanying Tobii software. Infants’ point-of-gaze were calibrated using a 5-point calibration scheme prior to data collection. The calibration scheme was repeated until an infant’s point-of-gaze was within 1° of the center of the target and repeated between the two trials. The video stimuli were presented only after the calibration criterion had been reached. Individual trials were removed from analyses due to failure to initially calibrate the infant’s eyes to the eye-tracking system (*N*_trials_ = 9) or failure to track an infant’s eyes because of excessive movement or fussiness (*N*_trials_ = 31).

Video frames were 8-bit color images and 720 by 480 pixels in resolution. Each frame was hand-traced for areas of interest, which were demarcated as a box encompassing each character’s face as in the prior studies of typical development using the same stimuli^34,51^. Fixations that fell within areas of interest were identified using software written in MATLAB (MathWorks, Inc; Natick, MA). The primary dependent measure was percent of fixations that were directed at the face areas of interest.

### Eye-tracking statistical analysis

Our primary interest from modeling the eye-tracking data was to estimate each infant’s rate of change in attention to faces from 3 to 12 months, such that individual developmental trajectories in social attention could be analyzed as a function of 6-week Salience Network connectivity. Longitudinal changes in percent fixation to faces were analyzed with a Bayesian hierarchical linear model in R (rstanarm package), which uses a Markov chain Monte Carlo simulation to draw a posterior distribution (e.g., a range of probable values for a variable given the data). This aspect of the Bayesian framework allows for greater precision in estimating parameters than by the frequentist approach^52,53^.

Attention to faces was operationalized as percent fixations to characters’ faces in each video stimulus. Developmental trajectories were modeled as the linear and quadratic effects of age (i.e., age and age^2^, respectively). ASD-likelihood group (HL versus TL) and video stimulus type (*Charlie Brown* versus *Sesame Street*) were modeled as fixed effects; age and intercept were modeled as random effects to account for individual differences and correlated repeated measures at 3, 6, 9, and 12 months. The inclusion of the change of slope (i.e., the quadratic term age^2^) aimed to capture the change in growth rate from 3 to 12 months. Student-t distributions were used as priors for the regression coefficients and standard deviation, and a Gaussian function was used as the identity link function.

The model in equation form is:

$${{{{{\rm{Level}}}}}}1:{{{{{\rm{Fixatio}}}}}}{{{{{{\rm{n}}}}}}}_{{{{{{\rm{ij}}}}}}}={{{{{\rm{intercep}}}}}}{{{{{{\rm{t}}}}}}}_{0{{{{{\rm{j}}}}}}}+{{{{{{\rm{B}}}}}}}_{1{{{{{\rm{j}}}}}}}{({{{{{\rm{Age}}}}}})}_{{{{{{\rm{ij}}}}}}}+{{{{{{\rm{B}}}}}}}_{2{{{{{\rm{j}}}}}}}{({{{{{\rm{Age}}}}}})}^{2}{{{{{\rm{ij}}}}}}+{{{{{{\rm{error}}}}}}}_{{{{{{\rm{ij}}}}}}}$$

$${{{{{\rm{Level}}}}}}2:{{{{{{\rm{Intercept}}}}}}}_{0{{{{{\rm{j}}}}}}}={\Upsilon }_{00}+{\Upsilon }_{01}({{{{{\rm{Likelihood\; Status}}}}}})+{\Upsilon }_{02}({{{{{\rm{Stimulus\; Type}}}}}})+{{{{{{\rm{error}}}}}}}_{0{{{{{\rm{j}}}}}}}$$

$${{{{{\rm{Level}}}}}}2:{{{{{\rm{B}}}}}}1{{{{{\rm{j}}}}}}={\Upsilon }_{10}+{\Upsilon }_{11}({{{{{\rm{Likelihood\; Status}}}}}})+{\Upsilon }_{12}({{{{{\rm{Stimulus\; Type}}}}}})+{{{{{{\rm{error}}}}}}}_{1{{{{{\rm{j}}}}}}}$$

$${{{{{\rm{Level}}}}}}2:{{{{{{\rm{B}}}}}}}_{2{{{{{\rm{j}}}}}}}={\Upsilon }_{20}+{\Upsilon }_{21}({{{{{\rm{Likelihood\; Status}}}}}})+{\Upsilon }_{22}({{{{{\rm{Stim\; Type}}}}}})+{{{{{{\rm{error}}}}}}}_{2{{{{{\rm{j}}}}}}}$$

Pareto k diagnostic values indicated a good model fit (all pareto k estimates were less that 0.7). Diagnostics regarding model fit and visualization of posterior distributions were verified with the shinystan package in R. Statistical significance was evaluated using two-tailed tests. As expected, there was a significant linear effect of age such that attention to faces increased with age across all participants (95% CI: [1.73 9.39]). The rate of change over time decreased across all infants indicating a quadratic trajectory in face-looking (95% CI: [−0.47 −0.01]). There was also a main effect of ASD-likelihood group such that HL infants overall attended less to faces than TL infants (95% CI: [−40.91 −3.66]).

Using the coef function in R, we extracted each infant’s estimate of developmental increase in face-looking from 3 to 12 months (i.e., each infant’s beta coefficient for the age term in the model). These estimates were then used as a covariate of interest in a model of Salience Network connectivity.

### MRI data acquisition

MRI data were acquired on a 3T Siemens Tim Trio scanner using a 12-channel head coil during natural sleep. Parents were instructed to put their infant to sleep using their normal bedtime routine. After the infant was asleep and swaddled, silicon earplugs were placed over the infant’s ear canal, and mini earmuffs were fitted over the entire outer ears. Infants were then placed on a custom-made bed that fit inside the scanner’s head coil and secured on the scanner bed with a Velcro strap. To minimize movement, a weighted blanket was used and foam pads were positioned around each infant’s head.

A localizer scan was used for graphic prescription. Structural matched bandwidth T2-weighted high-resolution echo planar images are acquired co-planar to the functional scans to ensure identical distortion characteristics to the fMRI scans (TR = 5000 ms, TE = 34 ms, matrix size 128×128, FOV = 192 mm, 34 slices, 1.5 mm in-plane resolution, with 4-mm-thick axial slices). Resting-state data were collected during an 8-min rs-fMRI scan (TR = 2000 ms, TE = 28 ms, matrix size 64 × 64, FOV = 192 mm, 34 slices, 3 mm in-plane resolution, with 4mm-thick axial slices).

### fMRI data preprocessing

Functional imaging data were preprocessed and analyzed using FSL version 5.0.8 (fMRIB’s Software Library^54^. Functional images were co-registered to the subject’s corresponding T2-weighted high-resolution anatomical scan, registered to an infant brain template^55^ using 12-parameter affine transformations, and spatially smoothed (Gaussian kernel of 6 mm FWHM) to increase signal-to-noise ratio. ICA-AROMA was used to detect and remove motion artifacts from the data^56^. This is a validated procedure that uses probabilistic independent component analysis to automatically detect participant-specific motion-related independent components while preserving signal of interest^57^. ICA-AROMA was selected over other motion denoising methods, such as deleting individual motion-contaminated volume (e.g., scrubbing)^58^ to effectively control for motion while maximizing data from the full scan. HL and TL infants did not differ in the average number of noise components identified by ICA-AROMA [HL: Mean = 27.25, SD = 9.43; TL: Mean = 27.72, SD = 11.37; *t(51)* = 0.16, *p* = 0.87, Cohen’s *d* = 0.05]; the number of noise components detected were comparable to that reported by Pruim and colleagues (23.1 components)^56^. HL and TL infants also did not differ on average relative motion prior to the denoising with ICA-AROMA (HL: Mean = 0.10 mm, SD = 0.07 mm; TL: Mean = 0.23 mm, SD = 0.82 mm; *t(51)* = 0.81*, p* = 0.42, Cohen’s *d* = 0.22). Data were then band-pass filtered (0.01–0.1 Hz). Nuisance regressors (e.g., mean cerebrospinal fluid, white matter, and global time series) from the bandpass filtered data were calculate and then regressed out of the filtered data to further remove noise. Given continuous debate in the field as per the pros and cons of implementing global signal regression^59^, group-level analyses were also completed without regressing global signal or its derivatives as nuisance variables; none of our main findings were affected by this change, including the inverse pattern of connectivity between frontal and sensorimotor regions (*r* = −0.428, *p* = 0.001, 95% CI: [−0.626 −0.178]).

### fMRI statistical analyses

Resting-state fMRI analyses were conducted with FSL fMRI Expert Analysis Tool (FEAT, version 6.0 www.fmrib.ox.ac.uk/fsl/^60^). Whole brain connectivity within the Salience Network was examined using a right anterior insula (rAI) seed^35^ that was derived from an anatomical parcellation of the right insula from a neonatal template. We identified the center of gravity of the right insula and created an anatomical mask composed of voxels anterior to the midline^55^. The center of gravity for our rAI seed (X = 23, Y = 5, Z = 1) corresponds to meta-analytic definitions of rAI^61^. Region-of-interest (ROI) time-series from each infant’s processed residuals in standard space were extracted and correlated with every other voxel in the brain to generate Salience Network functional connectivity maps. Individual Salience Network maps were converted into z-statistic maps using Fischer’s r-to-z transformation. At the group level, we modeled a 2-sample mixed-effects design, at *Z* > 3.1 with cluster correction for multiple comparisons at *p* < 0.05, using FSL FLAME (FMRIB’s Local Analysis of Mixed Effects State) Stage 1 + 2. Statistical significance was evaluated using two-tailed tests. For the between-group comparisons and regression analyses (see below), significance was assessed voxel-wise at *p* < 0.05, controlling for multiple comparisons using cluster-level correction estimated by AFNI’s 3dClustSim with 10,000 iterations at initial cluster forming thresholds of both *p* < 0.01 (*Z* = 2.3) and *p* < 0.001 (*Z* = 3.1), a mixed-model spatial autocorrelation function, and a joint [HL + TL] Salience Network connectivity map. Family income and gestational age were examined as potential covariates; as none contributed significantly they were excluded from the final analyses. Regression analyses with estimates of increases in face-looking from 3 to 12 months, AOSI, ESCS, and Infant/Toddler Sensory Profile were restricted to (i.e., masked by) the joint [HL + TL] Salience Network connectivity maps (see Table 2). Parameter estimates of connectivity strength for between-group comparisons were extracted from significant clusters using FMRIB and evaluated using Pearson correlation.

**Table 2:** Coordinates of regions with significant functional connections to the right anterior insula

Region	Side	Peak mm (x,y,z)	Max Z
SN connectivity map between group comparison: HL > TL			
Precentral Gyrus	L	(−14, −17, 40)	3.41
Postcentral Gryus	L	(−35, −15, 27)	3.25
SN connectivity map between group comparison: TL > HL			
Inferior Frontal Gyrus	R	(26, 11, 4)	3.58
Positive correlations between right anterior insula and Face-looking in TL group			
Orbitofrontal Cortex	R	(35, 21, −5)	4.61
Inferior Frontal Gyrus	R	(29, 8, 7)	4.36
Anterior Cingulate Gyrus	R	(11, 13, 10)	4.08
Positive correlations between right anterior insula and ESCS IJA in TL group			
Orbitofrontal Cortex	R	(33, 19, −4)	3.25
Positive correlations between right anterior insula and ITSP Sensory Sensitivity in HL group			
Superior Temporal Gyrus	R	(35, −4, 8)	3.85
Negative correlations between right anterior insula and AOSI Total Markers in HL group			
Putamen	L	(−14, 0 11)	4.10
Insula	L	(−19, 0, 10)	3.41
Hippocampus	L	(−16, −16, −2)	3.41

### Additional control analyses

To address the specificity of our findings to the Salience Network, additional analyses were conducted using posterior cingulate cortex, bilateral dorsolateral prefrontal cortex, and bilateral pre/postcentral gyri seeds to separately evaluate connectivity in the default mode network, the fronto-parietal attentional network, and sensorimotor networks, respectively, following the dame data-analytic procedures. Atypicalities in functional connectivity in the default mode network and fronto-parietal networks have been observed in youth and adults with ASD, thus serving as good control networks to examine potential developmental origins of disrupted network connectivity in individuals with ASD^15,62,63^. However, the default mode and fronto-parietal networks undergo substantive development during the first year whereas the somatosentory network is present at birth^64^. Accordingly, examining this network would allow to further evaluate whether the observed between-group differences in network connectivity between HL and TL infants are specific to the Salience Network or are more broadly reflective of disruptions in early functional brain organization^65^.

These additional seed-based connectivity analyses were implemented according to the same procedures as used for the Salience Network, as follows: Anatomically-defined seeds were used to identify the default mode network, fronto-parietal network, and sensorimotor networks for whole-brain connectivity analyses. The 5-mm seeds for these three networks were the precuneus (Montreal Neurological Institute coordinates; MNI: [−1, −30, 12]^66^; bilateral dorsolateral prefrontal cortex (MNI: [+/−30, 11, 25]), and bilateral pre- and post-central gyri (MNI: [+/−28, −11, 32]), respectively. FSLMATHS^54^ was used to identify the center of gravity (i.e., coordinates in MNI space) for the precuneus and pre-/post-central gyri based on the parcellation available for the infant anatomical atlas^55^ However, because the atlas does not include a separate dorsolateral prefrontal cortex region of interest, the meta-analytic database Neurosynth (https://neurosynth.org)^61^ was used to identify the MNI coordinates for the dlPFC seed.

As shown in Supplementary Fig. 1, we did not observe any significant between-group differences in connectivity across these three additional networks, attesting to the specificity of our findings of early atypicalities in SN connectivity in infants at high familial risk for ASD.

### Equivalence tests

To further qualify the non-significant between-group differences in connectivity within these three additional control networks, we conducted equivalence tests using the TOSTER package in R^67^. First, we used the FSL command fslmeants to separately extract parameter estimates of connectivity for HL and TL groups, from the respective within-group connectivity maps for each of these three control networks (DMN, frontoparietal, and sensorimotor networks). While effect sizes are not commonly reported in the functional connectivity literature, we calculated the standardized effect size associated with a Z score ≥3.1 and our sample size, which can be interpreted as the critical effect size. We used this value as the smallest effect size of interest (SESOI). Using this value, an equivalence test can reject effect sizes falling outside that bound. In this case, the critical effect size was *d* = 0.855.

The TOST (two one-sided test) procedure for Student’s equivalence test for independent samples, with equivalence bounds of ΔL = –0.855 and ΔU = 0.855, revealed that the effects observed were statistically equivalent across HL and TL groups for both the frontoparietal network (t(51) = −1.861, *p* = 0.0342) and sensorimotor network (t(51) = −2.183, *p* = 0.0168). Despite a trend in the same direction, this was not the case for the default mode network (t(51) = −1.286, *p* = 0.102), warranting further examination of this network in future studies.

### Statistics and reproducibility

Detailed explanations for statistical approaches used in all analyses (i.e., eye tracking, within- and between-group functional connectivity, correlations between functional connectivity and behavior, and control connectivity analyses) are provided in each of the relevant sections above. Our experiments were not replicated, but a description of power and effect size is described in the Participants section, and effect sizes and/or confidence intervals are provided for each analysis, as relevant.

### Reporting summary

Further information on research design is available in the Nature Portfolio Reporting Summary linked to this article.

### Supplementary information

Supplementary Information

Description of Additional Supplementary Files

Supplementary Data 1

Reporting Summary

## Data Availability

The raw data that support the findings of this paper are available via the National Institute of Mental Health Data Archive (NDA; https://nda.nih.gov/)^69^. Dataset identifier: 10.15154/qx4v-t626. The source data for Figs. 2c, 3a, 3c, and 4 are provided in Supplementary Data 1.

## Code availability

Eye movements and coding of fixation data were conducted with software written in MATLAB (MathWorks), available from Zenodo at this link: https://zenodo.org/record/8403701^68^.
