# Supplementary material for: Salience network connectivity is altered in 6-week-old infants at heightened likelihood for developing autism
Source: Commun Biol. 2024 Apr 22;7:485. doi: 10.1038/s42003-024-06016-9 (PMC11035613; doi:10.1038/s42003-024-06016-9)
Supplement: Supplementary file 1 — Supplementary Information [file 42003_2024_6016_MOESM1_ESM.pdf]

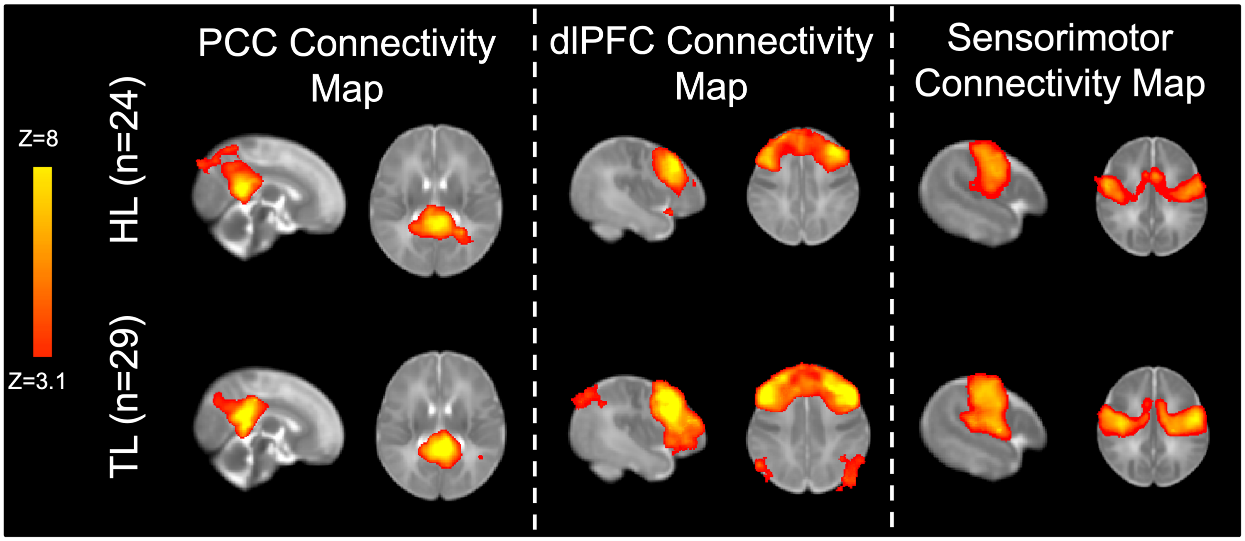

Supplementary Figure 1. Whole-brain connectivity maps using seed-based analyses from anatomically-defined posterior cingulate cortex (PCC), dorsolateral prefrontal cortex (dlPFC), and Sensorimotor seeds, respectively. We did not observe between-group differences in n=24 High Likelihood (HL) and n=20 Typical Likelihood (TL) infants.
