# Supplementary material for: Salience network connectivity is altered in 6-week-old infants at heightened likelihood for developing autism
Source: Commun Biol. 2024 Apr 22;7:485. doi: 10.1038/s42003-024-06016-9 (PMC11035613; doi:10.1038/s42003-024-06016-9)
Supplement: Supplementary file 2 — Description of Additional Supplementary Files [file 42003_2024_6016_MOESM2_ESM.pdf]

Description of Additional Supplementary Files

File name: Supplementary Data 1
Description: The source data for Figures 2c, 3a, 3c, and 4.
