# Supplementary material for: Salience network connectivity is altered in 6-week-old infants at heightened likelihood for developing autism
Source: Commun Biol. 2024 Apr 22;7:485. doi: 10.1038/s42003-024-06016-9 (PMC11035613; doi:10.1038/s42003-024-06016-9)
Supplement: Supplementary file 4 — Reporting Summary [file 42003_2024_6016_MOESM4_ESM.pdf]

nature research

Corresponding author(s): mirella@ucla.edu

Reporting Summary

Nature Research wishes to improve the reproducibility of the work that we publish. This form provides structure for consistency and transparency in reporting. For further information on Nature Research policies, see Authors & Referees and the Editorial Policy Checklist.

Statistical parameters

When statistical analyses are reported, confirm that the following items are present in the relevant location (e.g. figure legend, table legend, main text, or Methods section).

n/a	Confirmed	
☐	☒	The exact sample size (n) for each experimental group/condition, given as a discrete number and unit of measurement
☐	☒	An indication of whether measurements were taken from distinct samples or whether the same sample was measured repeatedly
☐	☒	The statistical test(s) used AND whether they are one- or two-sided<br>*Only common tests should be described solely by name; describe more complex techniques in the Methods section.*
☐	☒	A description of all covariates tested
☐	☒	A description of any assumptions or corrections, such as tests of normality and adjustment for multiple comparisons
☐	☒	A full description of the statistics including central tendency (e.g. means) or other basic estimates (e.g. regression coefficient) AND variation (e.g. standard deviation) or associated estimates of uncertainty (e.g. confidence intervals)
☐	☒	For null hypothesis testing, the test statistic (e.g. F, t, r) with confidence intervals, effect sizes, degrees of freedom and P value noted<br>*Give P values as exact values whenever suitable.*
☐	☒	For Bayesian analysis, information on the choice of priors and Markov chain Monte Carlo settings
☐	☒	For hierarchical and complex designs, identification of the appropriate level for tests and full reporting of outcomes
☐	☒	Estimates of effect sizes (e.g. Cohen's d, Pearson's r), indicating how they were calculated
☒	☐	Clearly defined error bars<br>*State explicitly what error bars represent (e.g. SD, SE, CI)*

Our web collection on statistics for biologists may be useful.

Software and code

Policy information about availability of computer code

Data collection	Imaging data were collected on a 3T Siemens Tim Trio scanner using a 12-channel head coil. Eye-tracking data were collected with a Tobii T60XL eye-tracker at 60 Hz with a spatial accuracy of approximately 0.5o accuracy. Eye-movements (e.g., fixations, blinks, and saccades) were detected using the accompanying Tobii software.
Data analysis	Functional imaging data were preprocessed and analyzed using FSL version 5.0.8 (fMRIB's Software Library). ICA-AROMA (Pruim et al., 2015) was used to detect and remove motion artifacts from the imaging data. Raw eye-tracking data were analyzed with custom software written in Matlab; statistical analyses were conducted using the rstanarm package in R.

For manuscripts utilizing custom algorithms or software that are central to the research but not yet described in published literature, software must be made available to editors/reviewers upon request. We strongly encourage code deposition in a community repository (e.g. GitHub). See the Nature Research guidelines for submitting code & software for further information.

April 2018

Data

Policy information about availability of data

All manuscripts must include a data availability statement. This statement should provide the following information, where applicable:

- Accession codes, unique identifiers, or web links for publicly available datasets
- A list of figures that have associated raw data
- A description of any restrictions on data availability

Coding of eye movements and fixation data were conducted with software written in MATLAB (MathWorks), available upon request from the corresponding author. The data that support the findings of this paper are available via the National Institute of Mental Health Data Archive (NDA; https://nda.nih.gov/).

Field-specific reporting

Please select the best fit for your research. If you are not sure, read the appropriate sections before making your selection.

☐ Life sciences ☒ Behavioural & social sciences ☐ Ecological, evolutionary & environmental sciences

For a reference copy of the document with all sections, see nature.com/authors/policies/ReportingSummary-flat.pdf

Behavioural & social sciences study design

All studies must disclose on these points even when the disclosure is negative.

Study description	The study was a longitudinal design. Infant participants were scanned at 6 weeks of age and prospectively followed during the first postnatal year. The data acquired were quantitative and measures were obtained through neuroimaging, eye-tracking, and behavioral methods.
Research sample	The research sample consisted of 53 infants at high versus typical likelihood for autism spectrum disorder (ASD). Participants in this study were enrolled as part of a longitudinal project examining early brain-based markers of ASD during the first year. The Institutional Review Board (IRB) at the University of California, Los Angeles, approved all protocols associated with the project, and all enrolled participants had informed consent provided by their parent/legal guardian. Infants were assigned to likelihood-based cohorts based on family history: high likelihood infants (HL) had at least one older sibling with a clinical ASD diagnosis whereas typical likelihood infants (TL) had no family history of ASD or any other developmental disorder. Prior research showed that the recurrence likelihood for developing ASD is approximately 20% in HL infants. Exclusionary criteria for both groups included: 1) indication of genetic or neurological conditions associated with ASD risk (e.g., fragile X syndrome, epilepsy, tuberous sclerosis), 2) significant perinatal insult or chronic medical conditions impacting development, 3) severe visual, hearing, or motor impairment, 4) non-English speaking parents, and 5) contraindication for MRI (e.g., metal implants). All participants were enrolled in the study prior to 6 weeks of age. HL and TL infants were matched by gender (Mann-Whitney $U = 306$, $p=0.38$), and birth weight ($t(51)=0.43$, $p=0.67$), as well as ethnicity and family socio-economic status (race: Mann-Whitney $U=341.5$, $p=0.88$; household income: Mann-Whitney $U=282.5$, $p = 0.23$). Given the diverse demographic of Los Angeles County, the research sample was representative of the broader population of infants with and without familial history of ASD.
Sampling strategy	High and typical likelihood families were broadly recruited in Los Angeles County. Our sample size was based on prior resting-state fMRI studies in infants. Based on effect sizes derived from these prior studies (e.g., Liu et al., 2008; Damaraju et al., 2014), a minimum sample size of 23 per group was sufficient to detect significant effects.
Data collection	Functional imaging data were preprocessed and analyzed using FSL version 5.0.8 (fMRIB's Software Library). ICA-AROMA (Pruim et al., 2015) was used to detect and remove motion artifacts from the imaging data. Eye-tracking data were collected using a Tobii T60XL eye-tracker at 60 Hz with a spatial accuracy of approximately 0.5o accuracy. Eye-movements (e.g., fixations, blinks, and saccades) were detected using the accompanying Tobii software. Observational-based behavioral measures were collected by a trained clinician and questionnaires about infant development were completed via pen and pencil by a parent.
Timing	Data were continuously collected between December 2012 through November 2017
Data exclusions	Neuroimaging data were excluded due to excessive head motion during scanning and/or scanner artifacts. Eye-tracking data were excluded due to failure to initially calibrate the infant's eyes to the eye-tracking system and/or failure to track an infant's eyes because of excessive movement or fussiness.
Non-participation	2 infants dropped out of the longitudinal study; no reason was provided by the family.
Randomization	Participants were grouped by likelihood for developing autism spectrum disorder (ASD). Given this parameter, randomization was not relevant, nor possible, for the current study.

Reporting for specific materials, systems and methods

Materials & experimental systems

n/a	Involved in the study
☒	☐ Unique biological materials
☒	☐ Antibodies
☒	☐ Eukaryotic cell lines
☒	☐ Palaeontology
☒	☐ Animals and other organisms
☐	☒ Human research participants

Methods

n/a	Involved in the study
☒	☐ ChIP-seq
☒	☐ Flow cytometry
☐	☒ MRI-based neuroimaging

Human research participants

Policy information about studies involving human research participants

Population characteristics	See above
Recruitment	Infant participants were broadly recruited throughout the Los Angeles County. IRB-approved fliers were posted in pediatrician offices, newspapers, magazines, community-based events, and the Center for Autism Research and Treatment at UCLA. IRB-approved recruitment messages were aired on Pandora and local radio stations. Word-of-mouth was also a means of recruitment. This ensured a broad sampling.

Magnetic resonance imaging

Experimental design

Design type	Resting-state fMRI
Design specifications	8-minutes continuous resting-state fMRI scan during natural sleep
Behavioral performance measures	Behavioral measures of social and cognitive development were measured outside of the scanner at 12 months (i.e., Autism Observation Scale for Infants, Mullen Scales of Early Learning, Early Social Communication Scale, Infant Toddler Sensory Profile)

Acquisition

Imaging type(s)	Functional connectivity resting-state fMRI
Field strength	3T
Sequence & imaging parameters	Spin-Echo, TR=2000ms, TE=28ms, matrix size 64x64, FOV=192mm, 34 slices, 3mm in-plane resolution, with 4mm-thick axial slices
Area of acquisition	Whole-brain

Diffusion MRI ☐ Used ☒ Not used

Preprocessing

Preprocessing software	FSL version 5.0.8 (FMRIB's Software Library) was used for preprocessing, which included image realignment, 4D mean intensity normalization, band-pass filtering (0.01Hz -0.1 Hz), and spatial smoothing (Gaussian kernel of 6mm FWHM). Skull-stripping of subjects' T2 anatomical image and mean functional image were performed using the brain extraction tool in FSL; if automated skull-stripping was unsatisfactory as determined by experts' visual inspection, raw images were manually skull-stripped using the brain surface extractor from BrainSuite. Co-registration and registration of images were performed using FMRIB's Linear Image Registration Tool (FLIRT). Artifact removal was performed using ICA-AROMA in FSL, which employs FSL's Multivariate Exploratory Linear Decomposition into Independent Components (MELODIC).
Normalization	All subjects' resting-state data were 4D mean intensity normalized by a single scaling factor ("grand mean scaling") as part of FSL's preprocessing procedure. Functional data co-registered to the subjects' high-resolution T2 anatomical scans were spatially transformed to match a neonatal template (Shi et al., 2011) via a 12 degree-of-freedom affine transformation.
Normalization template	Data from the subjects' native space were transformed into standard space using a neonatal atlas from Shi et al. (2011), which was developed from 95 infants.
Noise and artifact removal	Artifacts and noise were removed using ICA-AROMA (Pruim et al., 2015), which is a data-driven method to identify/remove components containing structured noise (e.g., head motion).

Volume censoring

ICA-AROMA procedures were completed in FSL following instructions and software that were made available by Pruim and colleagues. Average number of noise components identified by ICA-AROMA did not differ by risk status [High Risk: Mean=27.25, SD=9.43; Low Risk: Mean=27.72, SD=11.37; $t(51)=0.16$, $p=0.87$]; the number of noise components detected were comparable to that reported by Pruim and colleagues (23.1 components).

Statistical modeling & inference

Model type and settings

rs-fMRI data were analyzed using FSL's Expert Analysis Tool (FEAT). At the group level, we modeled a 2-sample mixed effects design with a 2-stage process using Bayesian modeling and estimation (FLAME 1+2) using Z>3.1, with correction for multiple comparisons at the cluster level p<0.01. Likelihood status was included as an explanatory variable (EV) in group-level analyses. Regression analyses examining the effects of social and cognitive development on salience network connectivity further included scores from behavioral measures as an additional EV in the linear model.

Effect(s) tested

We examined the effects of likelihood status (high and typical likelihood) as well as their interactions in the linear models.

Specify type of analysis: ☒ Whole brain ☐ ROI-based ☐ Both

Statistic type for inference
(See Eklund et al. 2016)

For the between-group comparisons and regression analyses, significance was assessed voxel-wise at p<.05, controlling for multiple comparisons using cluster-level correction estimated by AFNI's 3dClustSim with 10,000 iterations at an initial cluster forming threshold of p<0.001 (Z=3.1), a mixed-model spatial autocorrelation function, and a joint [high likelihood+typical likelihood] Salience network connectivity map. Between-group and correlational analyses were restricted to regions where high and typical likelihood groups showed significant Salience Network connectivity.

Correction

Cluster-level threshold controls for family-wise error rate (FWER).

Models & analysis

n/a	Involved in the study
☐	☒ Functional and/or effective connectivity
☒	☐ Graph analysis
☒	☐ Multivariate modeling or predictive analysis

Functional and/or effective connectivity

At the single subject level, functional connectivity between the right anterior insula and all other voxels in the brain was calculated using Pearson correlations. In line with established resting-state protocols, Pearson correlations with r > 0.4 were used to develop single-subject connectivity maps. These values were then transformed into Z values in order to make statistical inferences at the group level as described above.
